# Surface Modification of PDMS-Based Microfluidic Devices with Collagen Using Polydopamine as a Spacer to Enhance Primary Human Bronchial Epithelial Cell Adhesion

**DOI:** 10.3390/mi12020132

**Published:** 2021-01-26

**Authors:** Mohammadhossein Dabaghi, Shadi Shahriari, Neda Saraei, Kevin Da, Abiram Chandiramohan, Ponnambalam Ravi Selvaganapathy, Jeremy A. Hirota

**Affiliations:** 1 Firestone Institute for Respiratory Health–Division of Respirology, Department of Medicine, McMaster University, Hamilton, ON L8N 4A6, Canada; dabaghi.sharif@gmail.com (M.D.); saraein@mcmaster.ca (N.S.); chanda15@mcmaster.ca (A.C.); 2 Department of Mechanical Engineering, McMaster University, Hamilton, ON L8S 4L7, Canada; shahris@mcmaster.ca (S.S.); selvaga@mcmaster.ca (P.R.S.); 3 Department of Chemical Engineering, McMaster University, Hamilton, ON L8S 4L7, Canada; dak@mcmaster.ca; 4 School of Biomedical Engineering, McMaster University, Hamilton, ON L8S 4K1, Canada; 5 Division of Respiratory Medicine, Department of Medicine, University of British Columbia, Vancouver, BC V6H 3Z6, Canada; 6 Department of Biology, University of Waterloo, Waterloo, ON N2L 3G1, Canada

**Keywords:** microfluidic, polydopamine, collagen, polydimethylsiloxane (PDMS), organ-on-a-chip

## Abstract

Polydimethylsiloxane (PDMS) is a silicone-based synthetic material used in various biomedical applications due to its properties, including transparency, flexibility, permeability to gases, and ease of use. Though PDMS facilitates and assists the fabrication of complicated geometries at micro- and nano-scales, it does not optimally interact with cells for adherence and proliferation. Various strategies have been proposed to render PDMS to enhance cell attachment. The majority of these surface modification techniques have been offered for a static cell culture system. However, dynamic cell culture systems such as organ-on-a-chip devices are demanding platforms that recapitulate a living tissue microenvironment’s complexity. In organ-on-a-chip platforms, PDMS surfaces are usually coated by extracellular matrix (ECM) proteins, which occur as a result of a physical and weak bonding between PDMS and ECM proteins, and this binding can be degraded when it is exposed to shear stresses. This work reports static and dynamic coating methods to covalently bind collagen within a PDMS-based microfluidic device using polydopamine (PDA). These coating methods were evaluated using water contact angle measurement and atomic force microscopy (AFM) to optimize coating conditions. The biocompatibility of collagen-coated PDMS devices was assessed by culturing primary human bronchial epithelial cells (HBECs) in microfluidic devices. It was shown that both PDA coating methods could be used to bind collagen, thereby improving cell adhesion (approximately three times higher) without showing any discernible difference in cell attachment between these two methods. These results suggested that such a surface modification can help coat extracellular matrix protein onto PDMS-based microfluidic devices.

## 1. Introduction

The miniaturization of biomedical devices has been increasing in demand, warranting new fabrication approaches to produce such devices that can be used in various diagnostic and biological applications [1]. The use of polydimethylsiloxane (PDMS) has been evident in various biomedical applications due to its biocompatibility, low barriers to cost, and fabrication, especially for microfluidic applications where rapid prototyping and inexpensive prototyping can allow for long term usage [2]. PDMS possesses these beneficial properties for applications ranging from cell sorting to organ-on-a-chip devices. Organ-on-a-chip devices have attracted attention from pharmaceutical companies and academic laboratories due to the potential to emulate physiological phenomena at a micro-scale [3,4]. Organ-on-a-chip platforms can recapitulate the complexity of tissues and organs to some extent by combining cellular and extracellular cues in the chip. In addition to promising features such as providing tissue barriers and hydrodynamic forces that organ-on-a-chip devices offer, the inner surface of organ-on-a-chip devices can be coated with extracellular matrix (ECM) components to resemble the native cellular microenvironment and improve cellular adhesion [3,5,6,7,8].

The majority of organ-on-a-chip devices have been made of PDMS because of its unique characteristics. However, the PDMS surface of microfluidic devices needs to be tailored before the cell culture is introduced so that cells can uniformly adhere to the surface, grow, and proliferate. Although a conventional surface modification technique such as oxygen plasma [9] can be used to introduce hydroxyl groups onto a PDMS surface and thereby improve cell attachment, such a modification is not stable due to the hydrophobic recovery of PDMS, which requires the introduction of cell culture immediately following plasma treatment to obtain optimal cell attachment. Oxygen plasma treatment, even for a short time, leads to the formation of a thin SiO_x_ layer on PDMS. This can result in crack formation, which may not be stable or render the PDMS surface in an undesired way [10]. Without oxygen plasma treatment, the PDMS surface remains hydrophobic and does not allow the cells to adhere and proliferate either [10]. Generally, ECM proteins enhance cell attachment and proliferation by interacting via integrins or other cell adhesion molecules presented on a cell’s membrane [11,12,13,14,15]. This means that cell receptors recognize the sequence of polypeptide chains in collagen molecules [16]. Another approach is to coat an ECM-based component such as collagen or fibronectin by physical adsorption that occurs as the result of weak electrostatic and van der Waals interactions between the ECM-based material and PDMS. This method has been followed since the earlier works in the field of microfluidics and organ-on-a-chip to improve cell adhesion [17,18]. Since the attachment between the ECM-based component and PDMS is physical, it may degrade over time and compromise the homogeneity of cultured cells. A more promising approach is to covalently bond ECM proteins to PDMS using a spacer or linker such as (3-aminopropyl)triethoxy silane (APTES) [19]. The use of silane-based materials has two disadvantages: First, it requires an oxygen plasma treatment to introduce hydroxyl groups onto PDMS prior to silane coating, which may not be easily accessible. Second, silane molecules are toxic and may cause cell death if some regions of the surafce are not fully covered by ECM proteins.

To capture the benefit of ECM protein coating while addressing the shortcomings of other surface modification techniques and to graft an ECM protein or a combination of them on PDMS, a simple method is used to coat polydopamine (PDA) onto PDMS as a biomolecule agent. Dopamine (DA) in an alkaline solution (pH = 8.5) undergoes oxidative polymerization and forms PDA layers on submerged surfaces [20]. PDA itself [21] or as a coating agent [10] can be utilized to improve cell adhesion on PDMS. In fact, PDA, as a linker, can interact with the amine groups of ECM proteins and covalently bind them onto the surface [22]. Though PDA coating has been widely used to render various substrates [22,23,24], including PDMS [21,25,26], for improved cell attachment, it has not been used to coat an ECM-based protein in a microfluidic device. In a recent study, it has been demonstrated that PDA coating inside a microfluidic device is stable enough to be stored for months at room temperature or can be sterilized under extreme conditions without any negative impact on cell viability and functionality [26]. Although it was shown that PDA coating would enhance the cell attachment, PDA cannot communicate and interact with cells in the way that ECM-based materials may.

## 2. Materials and Methods

### 2.1. Device Fabrication 

PDMS microfluidic devices were produced using a Sylgard^®^ 184 silicone elastomer kit (purchased from Ellsworth Adhesives Canada, Hamilton, ON, Canada; Manufactured by Dow Inc. Midland, MI, USA) mixture at a 10:1 ratio of base and cross-linker agent. It was degassed for 30 min, cast onto a silicon wafer mold (two silicone tubes were placed in the designated locations as the inlet and outlet before pouring PDMS), and allowed to cure for at least an hour on a hot plate or in an oven at 85 °C. After curing, the casted PDMS devices were removed and they were then oxygen plasma or flame [27] treated with another layer of PDMS (oxygen plasma treatment was performed for two minutes at a pressure of 900 mTorr in a Harrick Plasma cleaner machine). After exposure, the microfluidic channel and the blank PDMS layer were brought into contact to instigate the bonding. The device could then be set to cure on a hot plate or in an oven at 85 °C for 24 h.

### 2.2. Polydopamine (PDA) Coating 

Dopamine hydrochloride was purchased from Sigma-Aldrich (Ontario, ON, Canada) and prepared at a 1, 2, and 5 mg mL^−1^ concentrations in 8.5 pH phosphate-buffered saline (PBS). Upon stirring, devices to be coated were connected in series with Tygon tubing. PDA solution was run through devices at various flow rates (0.5, 1, 3, 6, and 9 mL min^−1^) with a peristaltic pump for 24 or 48 h. For static PDA coating, the microfluidic devices were filled with the DA solution at the desired concentration for 24 or 48 h. After PDA coating, the devices were rinsed with PBS (pH = 7.4) and Deionized (DI) water.

### 2.3. Contact Angle Measurement and Atomic Force Microscopy (AFM)

After PDA coating and rinsing, the microfluidic devices were cut along the length of the channel to expose the inner coated surfaces to the atmosphere. Subsequently, the samples were dried at room temperature overnight prior to surface analysis measurements. For contact angle measurements, a 2 µL water droplet was used.

For AFM, we used a silicon nitride cantilever that had a spring constant of 0.4 N m, and it was adjusted to automatically scan the treated and non-treated surfaces at a scan rate of 1 Hz by acquiring 512 samples per line. The instrument software (NanoScope Analysis) was utilized for image analysis.

### 2.4. Cell Culture and Calcein AM Imaging

Human ethics: All studies using primary human lung material were approved by the Hamilton Integrated Research Ethics Board (5099-T).

Primary human airway epithelial cells: Primary human bronchial epithelial cells (HBECs) were isolated immediately from bronchial brushing into T25 flasks containing Pneumacult™ Ex-Plus Basal Media (Stemcell Technologies, Vancouver, BC, Canada) with Pneumacult™ Ex-Plus 50x Supplement, 0.01% hydrocortisone stock solution, and 1% antibiotic–antimycotic. Once cultures achieved ~80% confluence, cells were passaged to a T75 flask. Cells were fed with Pneumacult™ Ex-Plus Basal Media on a two-day feeding cycle. Once cultures reached 100% confluency, cells were trypsinized and seeded into microfluidic devices. A syringe pump was used to feed cells cultured in microfluidic devices every day. The feeding flow rate was 200 µL min^−1^, the devices were fed for 5 min, and the supernatant was collected for further analysis.

Calcein AM (5 µM, invitrogen, Carlsbad, CA, USA; purchased from Thermo Fisher with the product number of C3100MP, Ottawa, ON, Canada) dye was used to conduct a quantitative viability assay on Day 7. First, the medium was removed from all microfluidic devices, and the cells were gently rinsed with warmed PBS using a 10 mL syringe. Next, 400 µL of Calcein AM were added to each microfluidic device, they were incubated with Calcein AM dye at 37 °C for 20 min, the dye was removed, the microfluidic devices were rinsed with warmed PBS using a 10 mL syringe, and they were imaged by an EVOS M7000 microscope (Thermo Fisher, Canada).

### 2.5. Coating Optimization

**Scheme 1:**
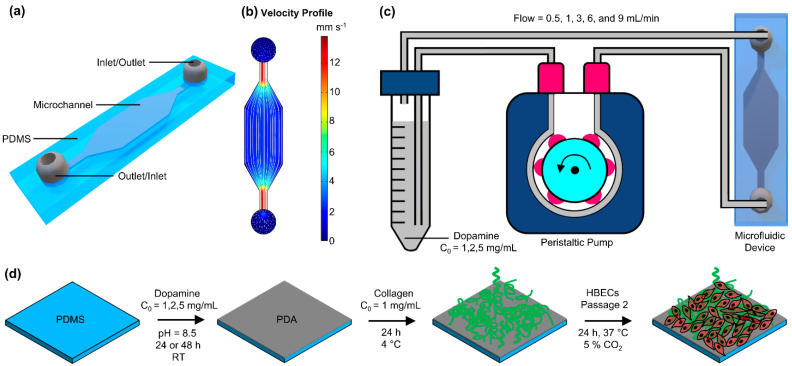
Representation of microfluidic device and the coating process: (**a**) 3D schematic view of the microfluidic device, (**b**) the velocity profile for the microfluidic device at a flow rate of 0.5 mL min^−1^ with flow streamlines showing the uniformity of flow inside of the device, (**c**) the polydopamine (PDA) dynamic coating process using a peristaltic pump, and (**d**) the coating process of PDA and collagen.

In this work, we developed and optimized PDA coating for microfluidic devices (Scheme 1a,b), where the coating was used as a linker to bind collagen inside the device. The height of the microfluidic channel was designed to be tall enough (~550 µm) to allow for further analysis of coated surfaces by cutting the channel and exposing the inner surfaces. Two methods were developed to coat PDA in microfluidic devices: (1) A simple one-step process in which DA solution was injected through the device and stored at room temperature for 24 or 48 h (static coating) and (2) DA solution was continuously perfused through microfluidic devices in a closed loop (dynamic coating), as shown in Scheme 1c. Scheme 1d represents the coating process and conditions for PDA and collagen coating.

## 3. Results and Discussion

**Figure 1:**
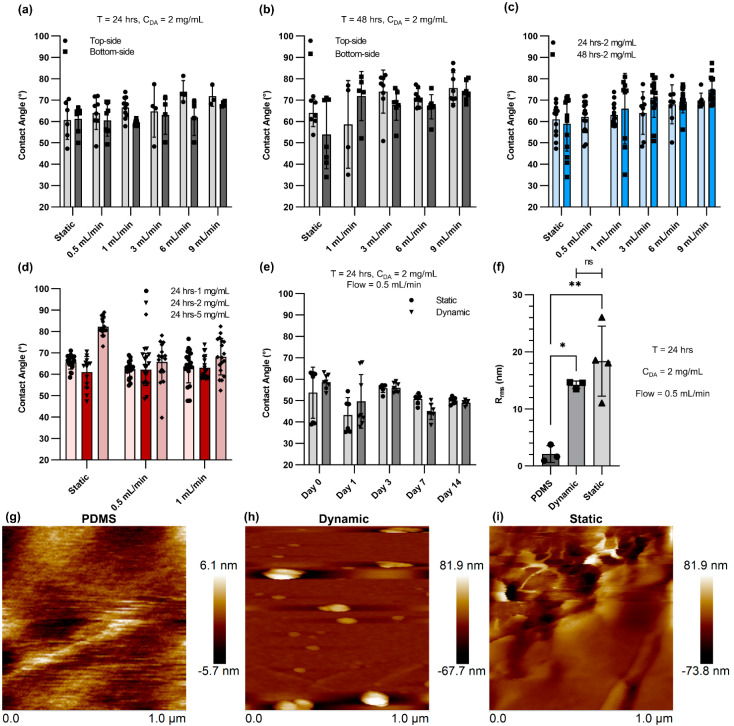
Characterization of PDA for microfluidic device: (**a**) Contact angle data for static PDA coating and dynamic PDA coating with different flow rates after 24 h coating with a concentration of 2 mg mL^−1^, (**b**) contact angle data for static PDA coating and dynamic PDA coating with different flow rates after 48 h coating with a concentration of 2 mg mL^−1^, (**c**) comparison of contact angle data for 24 h and 48 h coating of PDA, (**d**) the effect of DA concentration on contact angle measurement for static and dynamic coating, (**e**) contact angle data for static PDA coating and dynamic PDA coating (flow rate = 0.5 mL min^−1^) over 14 days tested under continuous flow, (**f**) surface roughness comparison of various conditions measured by atomic force microscopy (AFM), (**g**) surface topography of a native PDMS surface, (**h**) surface topography of a PDMS surface coated with PDA using dynamic coating technique (flow rate = 0.5 mL min^−1^), and (**i**) surface topography of a PDMS surface coated with PDA using static coating method.* *p* ≤ 0.05 and ** *p* ≤ 0.01.

In the beginning, the effect of flow rate, DA concentration, and duration of the coating was investigated by measuring the water contact angle of the PDMS-coated devices. The aim of the characterization step was to optimize the coating recipe for a microfluidic device. In the first step, the impact of coating time under a constant DA concentration of 2 mg mL^−1^ was studied (Figure 1a,b). The water contact angle for the top side and the bottom side was separately measured to ensure that the coating was uniform and resulted in similar hydrophilicity. Generally, the water contact angle varied between ~50° and ~70°, and the measurements among the various conditions were not significantly different. However, an increase in coating time led to larger variation between the top and bottom sides of the channels, showing that some non-uniformity or aggregation of nanoparticles could occur [28]. Figure 1c displays the average water contact angle for both 24 h and 48 h of PDA coating. The water contact angle was gently raised insignificantly by increasing the flow rate. However, the data were distributed closely around the average water contact angle when the coating time was 24 h and there was a flow rate of 0.5 mL min^−1^ or 1 mL min^−1^ for dynamic coating. Figure 1d shows the average water contact angle for static and dynamic conditions (flow rate = 0.5 mL min^−1^ and 1 mL min^−1^; time = 24 h) with three different initial DA concentrations. Higher DA concentrations led to a small increase in the average water contact angle, particularly for the static condition, and larger variation in the data distribution. Therefore, the DA concentration was chosen to be 2 mg mL^−1^ for the rest of the study, and the flow rate was fixed at 0.5 mL min^−1^ for dynamic coating. Next, the stability of PDA coating for both static and dynamic conditions was assessed by connecting the coated devices to a closed-loop fluid circuit in which DI water continuously flowed at a flow rate of 10 mL min^−1^. The average water contact angle was measured at Day 0, 1, 3, 7, and 14 and did not show any noticeable change, suggesting that the PDA coating was stable (Figure 1e). The roughness and topography of various treated and non-treated PDMS surfaces with PDA were also analyzed using AFM. Figure 1f depicts the root mean square (RMS) roughness of non-treated PDMS surfaces (native) and treated PDMS surfaces with PDA coating using the static and dynamic coating methods. The RMS roughness of native PDMS surfaces was 2.108 nm, which can be considered a typical measurement for PDMS. Nonetheless, the RMS roughness of PDA-coated PDMS surfaces increased to 14.267 nm and 18.375 nm for the dynamic and static methods, respectively. This likely occurred due to the aggregation of PDA molecules on the PDMS surface, which could result in the formation of PDA nanoparticles [29,30]. The surface topography of the non-treated PDMS surface and the PDMS surfaces treated with PDA coating (static and dynamic) are presented in Figure 1g–i.

**Figure 2:**
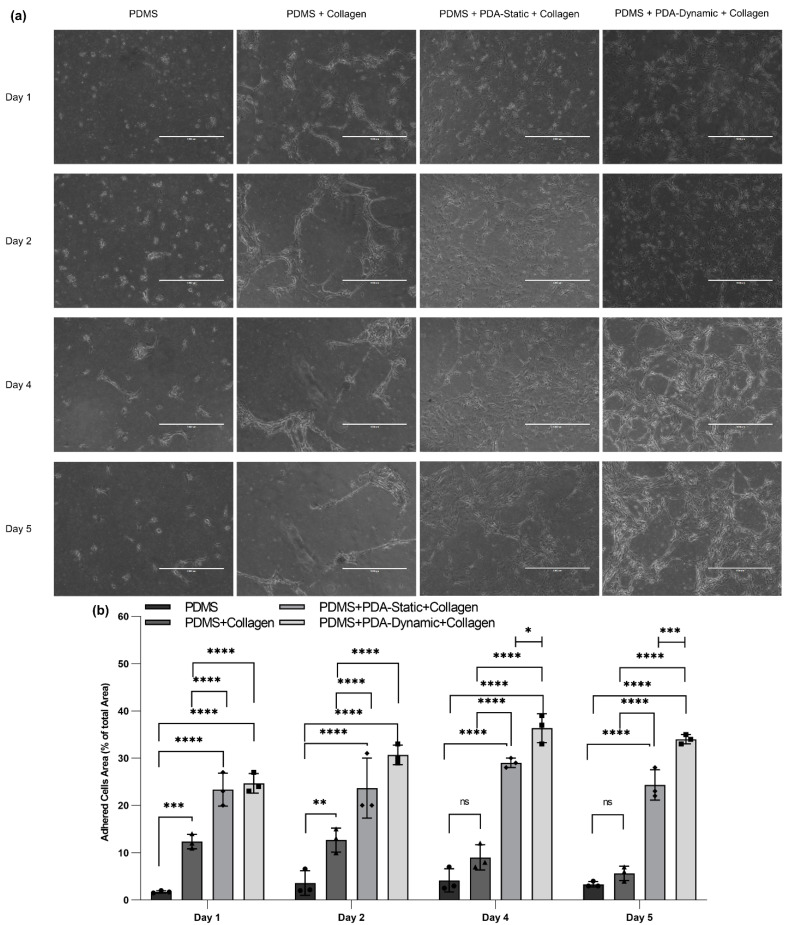
The comparison of cultured human bronchial epithelial cells (HBECs) on PDMS and treated surfaces: (**a**) Bright-field image of HBECs adhered on native, collagen-coated, PDA-static-collagen-coated, and PDA-dynamic-collagen-coated surfaces at Day 1, 2, 4, and 5 and (**b**) the adhered cells area as a percentage of the total area using bright field images that were quantified with ImageJ software. Scale bars are 1000 µm. * *p* ≤ 0.05, ** *p* ≤ 0.01, *** *p* ≤ 0.001, and **** *p* ≤ 0.0001.

Primary human bronchial epithelial cells (HBECs) were cultured (cell density was 1.5 × 10^5^ per device) in untreated and treated devices to evaluate the biocompatibility of various coating conditions compared to native PDMS, as seen in Figure 2a. Moreover, the covered surface areas of adherent HBECs were quantified as a percentage of the total surface area using ImageJ, as shown in Figure 2b. HBECs on non-treated PDMS devices exhibited the lowest attachment (~2%) and the lowest growth over five days, confirming that native PDMS had poor surface biocompatibility for HBECs. In contrast, the cells on treated PDMS devices coated physically with collagen displayed a higher attachment and density compared to those on native PDMS surfaces. However, the adherent cell area for collagen-treated PDMS devices decreased from Day 1 to Day 5, showing lifting of cells. The cells adhered at a higher density to the other two collagen–PDA-coated devices, and they slowly spread over five days. On Day 1 and Day 2, the adherent cell density between the two conditions (PDMS + PDA-Static + Collagen and PDMS + PDA-Dynamic + Collagen) was not significantly different. On Day 4 and Day 5, a discernible difference between the adherent cell density was observed, suggesting that the PDA-dynamic coating could provide a more suitable surface for cell proliferation. This observation is supported by the AFM results. The static PDA coating could lead to an increased formation of PDA nanoparticles compared to the dynamic PDA coating, thereby increasing the surface roughness.

**Figure 3:**
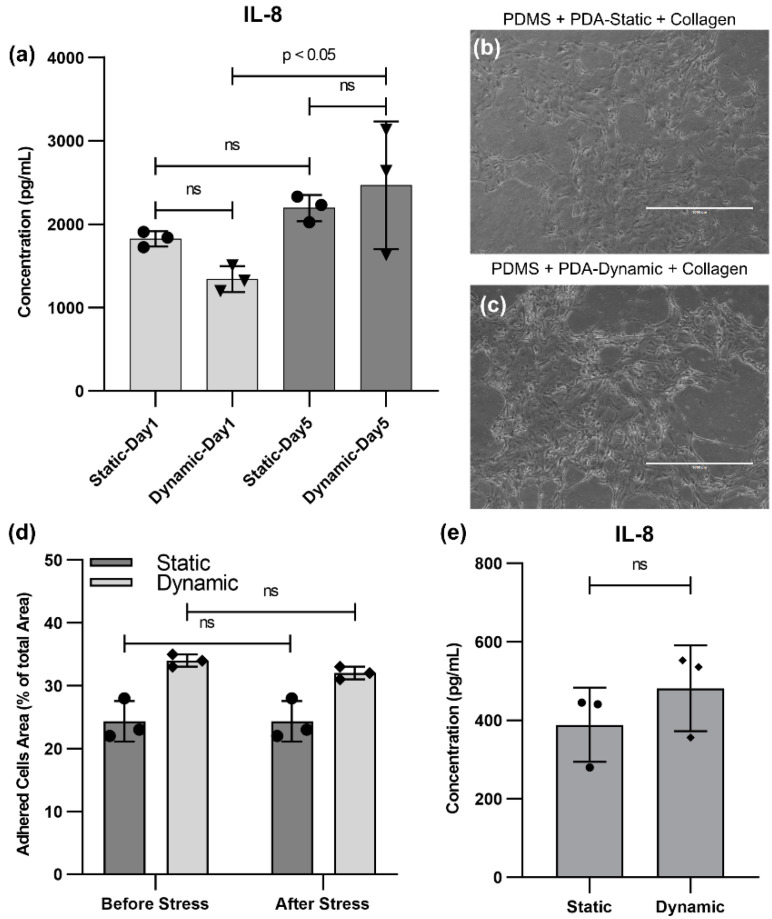
HBEC conditions before and after flow stress test: (**a**) IL-8 cytokine production by HBECs before flow stress at Day 1 and Day 5 for PDMS-treated surfaces with collagen after dynamic or static PDA coating, (**b**) a bright field image of HBECs on a PDMS-treated surface with collagen using static PDA coating after the flow stress test, (**c**) a bright field image of HBECs on a PDMS-treated surface with collagen using dynamic PDA coating after the flow stress test, (**d**) a comparison of the adhered cells area as a percentage of the total area before and after flow stress testing for both dynamic and static PDA coating, and (**e**) IL-8 cytokine production by HBECs after flow stress for PDMS-treated surfaces with collagen after the dynamic and static PDA coating.

The cells were challenged by a flow stress test where the cells were exposed to a high flow rate for one minute. This sudden increase in shear stress was used to challenge the adhesion of cells to the surface. Moreover, a weaker collagen bonding would be prone to degradation, thereby detaching cells from the surface. Next, their viability and interleukin 8 (IL-8) (a marker of inflammation routinely studied in epithelial cell experiments [31,32,33]) were measured before and after the stress to explore the effect of the PDA coating method on cell viability and functionality. Since the cells did not adhere properly to native PDMS devices and collagen-coated PDMS devices (without PDA coating), these two groups were excluded from further studies. Next, we compared the static and dynamic PDA coating using the flow stress test. The production level of IL-8 on Day 1 and Day 5 (before the flow stress test) was measured, as shown in Figure 3a. No perceptible difference between the two conditions was observed. Nevertheless, the production of IL-8 for devices with the dynamic PDA coating on Day 5 was significantly higher than Day 1. The cell density for the dynamic PDA-coated samples was increased by 40% from Day 1 to Day 5, while the cell density for the static PDA-coated samples only exhibited 5% growth. As a result, the increase in IL-8 production have could occured because there were more viable cells on Day 5 for the dynamic PDA-coated samples compared to Day 1. During the flow stress test, the flow rate was increased to 10 mL min^−1^ for one minute and the supernatant was collected for further measurement, after feeding the devices at a normal flow rate of 200 µL min^−1^. Optical microscopy was performed to examine the samples (Figure 3b,c), confirming that the cells still adhered to the surface. Figure 3d exhibits the adhered cell density before and after flow stress for both static and dynamic PDA-coated devices and shows no significant change. Moreover, the production of the IL-8 cytokine was evaluated after the flow stress test to examine the effect of the coating type on the inflammatory response to stress (Figure 1e). No significant difference between the two PDA coatings was observed, suggesting that the type of coating did not play a role in the production level of the inflammatory cytokine IL-8 during the flow stress test.

**Figure 4:**
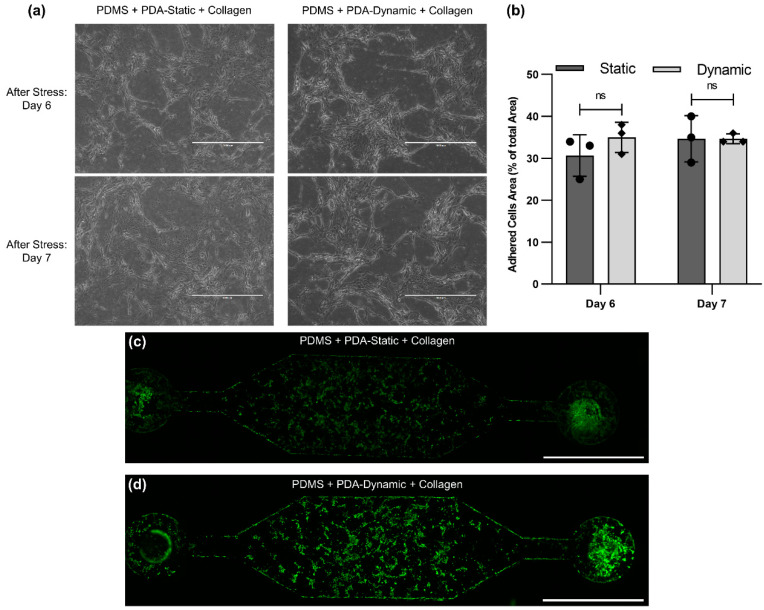
HBECs after flow stress test: (**a**) Bright-field image of HBECs adhered on PDA-static-collagen-coated and PDA-dynamic-collagen-coated surfaces at Day 6 (one day after flow stress test) and Day 7 (two days after flow stress test), (**b**) the adhered cell area as a percentage of the total area at Day 6 and Day 7 for static and dynamic conditions, and a fluorescence image of HBECs adhered in a PDMS-treated microfluidic device using (**c**) PDA-static-collagen coating and (**d**) PDA-dynamic-collagen coating.

After the flow stress test, devices with PDA and collagen coating were maintained for two additional days to ensure that the cells adhered to the surface, as displayed in Figure 4a. For both static and dynamic coating, the cell density did not change after the flow stress test (Figure 4b) and no significant difference between the static and dynamic coating was observed. On Day 7, the samples were stained with Calcein AM and imaged, as seen in Figure 4c,d. The images demonstrated that the cells were alive and were not impacted by the flow stress test. Moreover, the cell density at the inlet and outlet of devices, where velocity and shear stress are expected to be higher, was instead lower.

## 4. Conclusions

In summary, this work presented and compared a static and dynamic PDA coating for microfluidic devices. Different parameters of these static and dynamic PDA coatings were studied and optimized by measuring the water contact angle and obtaining AFM images. PDA coating was used to bind collagen inside the microfluidic devices to improve the cell adhesion of primary human bronchial epithelial cells. Cell density, cytokine production, and resistance to a flow stress test were used to compare the effect of the coating method on the cells. Cells could adhere properly to PDA-collagen-coated surfaces using both coating methods and no significant difference between these two methods was observed. These results suggest that either the static or dynamic PDA coating can be applied to tailor PDMS-based microfluidic devices for binding ECM proteins. Such surface modification can have an application in micro-scale cell culture systems or organ-on-a-chip devices where stable, long-term surface modifications are sought.

## Data Availability

The data that support the findings of this study are available from the corresponding author upon reasonable request.
